# Algal glycobiotechnology: omics approaches for strain improvement

**DOI:** 10.1186/s12934-021-01656-6

**Published:** 2021-08-21

**Authors:** Ranjna Sirohi, Jaemin Joun, Hong II Choi, Vivek Kumar Gaur, Sang Jun Sim

**Affiliations:** 1 Department of Chemical & Biological Engineering, Korea University, Seoul, 136713 Republic of Korea; 2 Amity Institute of Biotechnology, Amity University Uttar Pradesh, Lucknow Campus, Lucknow, 226 001 India

**Keywords:** Omics, Microalgae, Genomics, Transcriptomics, Proteomics, Metabolomics

## Abstract

Microalgae has the capability to replace petroleum-based fuels and is a promising option as an energy feedstock because of its fast growth, high photosynthetic capacity and remarkable ability to store energy reserve molecules in the form of lipids and starch. But the commercialization of microalgae based product is difficult due to its high processing cost and low productivity. Higher accumulation of these molecules may help to cut the processing cost. There are several reports on the use of various omics techniques to improve the strains of microalgae for increasing the productivity of desired products. To effectively use these techniques, it is important that the glycobiology of microalgae is associated to omics approaches to essentially give rise to the field of algal glycobiotechnology. In the past few decades, lot of work has been done to improve the strain of various microalgae such as *Chlorella*, *Chlamydomonas reinhardtii, Botryococcus braunii* etc., through genome sequencing and metabolic engineering with major focus on significantly increasing the productivity of biofuels, biopolymers, pigments and other products. The advancements in algae glycobiotechnology have highly significant role to play in innovation and new developments for the production algae-derived products as above. It would be highly desirable to understand the basic biology of the products derived using -omics technology together with biochemistry and biotechnology. This review discusses the potential of different omic techniques (genomics, transcriptomics, proteomics, metabolomics) to improve the yield of desired products through algal strain manipulation.

## Background

The term ‘glycobiotechnology’ term was coined to define an interdisciplinary research area that provided a classical understanding of the structure-function relationships of glycoconjugates. Here, glycoconjugates majorly refers to the interaction of complex carbohydrates with proteins (glycoproteins) and lipids (glycolipids) and also covers various glycosylated structures including hormones, antibiotics, proteoglycans and other metabolites [1, 2]. Glycoconjugates play an important role in inter- and intracellular communication. The study of these glycoconjugates is popularly known as glycomics, which is a subset of glycobiology and explains how a diverse structure of the glycan participates in biochemical processes. Glycobiotechnology is well described in various eukaryotes such as vertebrates, plants and insects, however, very few studies have been undertaken to investigate the glycobiotechnology in algae.

Algae and cyanobacteria represent a polyphyletic group that includes numerous species such as blue-green algae (*Arthrospira, Synococcous*), chlorophytes or green algae (*Chlorella, Chlamydomonas*) and heterokonts (diatoms). The diversity of algae results in extraordinary pathways offering large possibilities for the production of value added compounds such as pigments, cosmetics, nutraceuticals, pharmaceuticals, lipids, biofuels and biopolymers which attract the interest of industries towards algae. Recently, many algal species have received industrial attention because of their capability to produce long chain fatty acids [Poly unsaturated fatty acids, (PUFA), omega-3- fatty acid], chlorophyll, carbohydrates, vitamins and other pigments like astaxanthin [3, 4] enabling extensive applications in the food, pharmaceutical and nutraceutical industries. In the last decade, the number of scientific publications on microalgae applications has increased from 410 to 3193 (increase of more than 678%). The exponential increase in publications on ‘microalgae application’ from 2010 to 2020 proves that researchers and industries are attracted towards the utilization of microalgae. Further reasons for the choice of algae include its fast growth rates or industrial growth rates, and sustainable production of fine chemicals and biofuels. In addition, algae are attractive expression systems for the sustainable bioproduction of a range of high value products (therapeutic proteins, pharmaceuticals etc.) [5]. Moreover, algae have the ability to tolerate wide range of environmental stress.

**Fig. 1:**
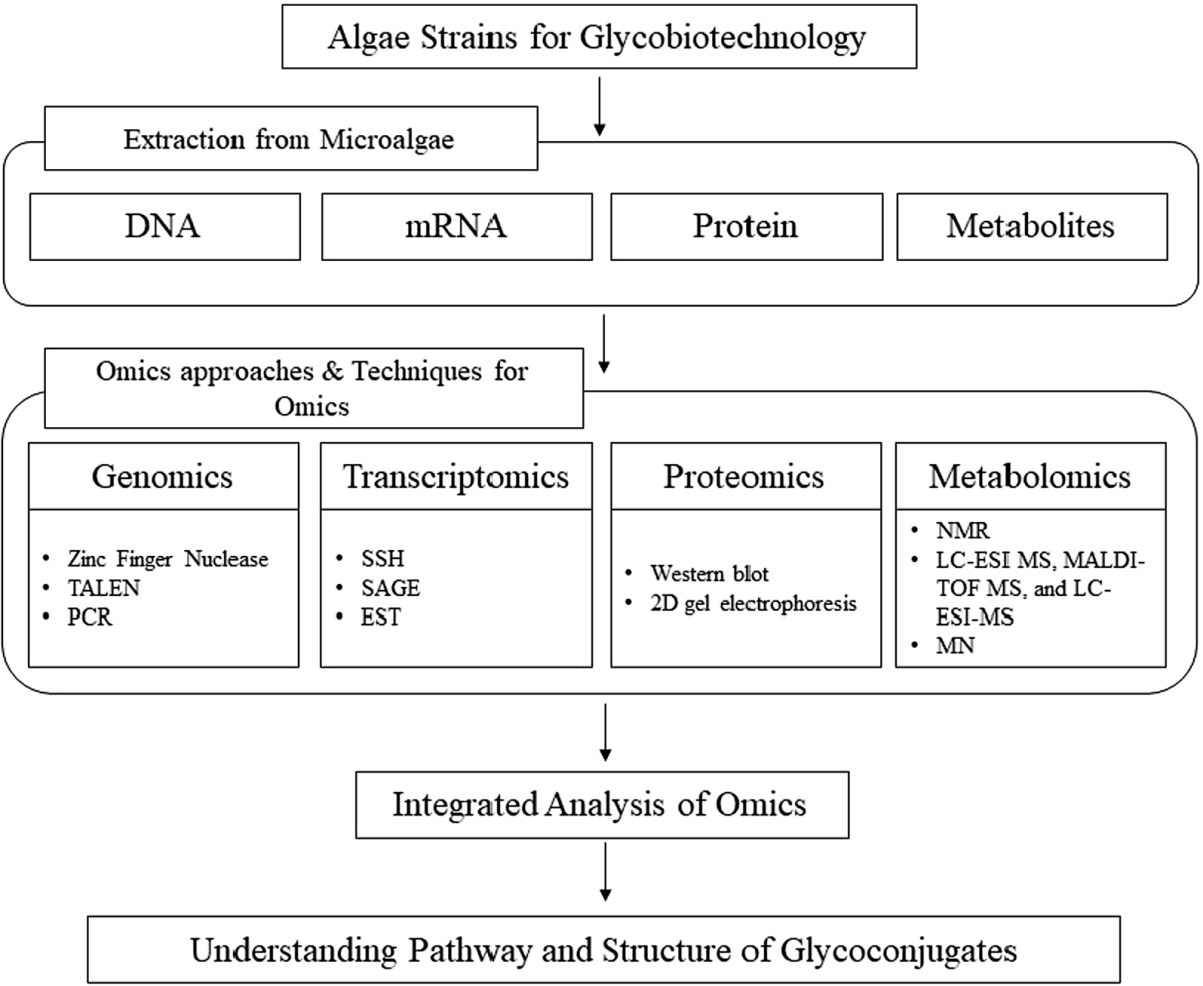
Workflow of analysis of glycoconjugates in microalgae via omics

Strain improvement in algae is becoming a promising strategy for the industries to increase the production of high market value products. Generally, industries use random mutagenesis and spontaneous mutation followed by intelligent screening process to construct an improved strain with desired properties. In the past, increasing production of the target molecule(s) was achieved using traditional techniques such as media manipulation, cultural techniques, improved downstream and extraction processes and, random mutagenesis by physical, chemical and ultraviolet methods [6]. In several cases, metabolic engineering have been successful for the strain improvement however, strain improvement of algae through metabolic engineering is still at an early stage and requires substantial development [7]. In this regard, the use of ‘omics’ approaches (genomics, proteomics, metabolomics, transcriptomics) will provide an in-depth understanding of the underlying biological processes (especially glycobiology) in algae that will provide new opportunities for algal strain development [8] (Fig. 1). Studies on the distribution of glycoconjugates such as lectin binding sites and lectin receptors at the algal cell surfaces would be highly relevant in this regard.

Algal glycobiology relates with the understanding broadly of structural and functional relationships of carbohydrates (and their derivatives), which are critical for the biological processes such as cellular and molecular and communications, governing the malfunction to control the disease and immunity and other aspects of developmental biology. From this point of view, via glycomics the complex information is conveyed by these biomolecules. Thus, the understanding the molecular mechanisms of the algae-derived products are critical. It is hoped that -omics studies will be helpful in providing the insights towards understanding the metabolic functions and possible solutions for developing algal biorefinery.

The advancements in algae glycobiotechnology have highly significant role to play in innovation and new developments for the production algae-derived products. It would be highly desirable to understand the basic biology of the products derived using -omics technology together with biochemistry and biotechnology. This would need screening and characterization of biomolecules employing -omics tools to reconstruct the metabolic pathways and remodeled process engineering to produce the products in high titers with desired properties. The cost of production will also be an important aspect. Such approaches on cellular and molecular mechanisms of algae-derived products are expected to result in efficient algal cell factories.

The benefits of algal strain improvement can be better understood through some recent advancements and applications. For instance, it is known that the cost of biofuels produced from algae is high as compared to petroleum due to its high processing cost which indicates that further improvements are required to make it economically feasible with provision forscale-up.The main reasons for this have primarily been low biomass and low lipids yields by the algal cultures explored.To increase the cell biomass and lipid contents in algae cell, metabolic and genetic engineering have been considered useful to target the pathways for triggering specific enzymes and modify specific genes appropriately to achieve the desired products [9]. For instance, *Phaeodactylum tricornutum* can be metabolically engineered by the overexpression of glycerol-3-phosphate acycltransferase 2 enzyme isoform in the algae for increasing the lipid content while decreasing the carbohydrate and protein content [10]. It was reported that 2.9-folds increase in triacylglycerides (preferred feedstock for biofuel) can be achieved as compared to glycolipids. In another investigation, the over expression of Acetyl-CoA (ACS) gene in *C. reinhardtii* increased the lipid content by 6-foldsas compared to the natural strain [11]. ACS and malonyl-CoA are the two desirable enzymes for lipid production that convert acetate to acetyl-CoA which is an important molecule in lipid synthesis. Apart from biofuels, the production of astaxanthin (a keto-carotenoid pigment) can also be enhanced by altering the algal glycobiology through strain manipulation.

Astaxanthin has anti-inflammatory and antioxidant activity and also shows therapeutic potential against Alzheimer, Parkinson, cancer and cardiovascular diseases [12]. Astaxanthin pigment is commonly produced by *Haematococcus pluvial* strain, cyanobacteria *Synechocystis* sp. (which is a prokaryote) does not express β-carotene gene naturally, and responsible enzyme has been engineered using DNA from *Haematococcus pluvialis* through cpc560 and psbA2 promoter [13]. Natural strain of *C. reinhardtii* is unable to produce pigments but after genetic engineering, this strain produced 1 mg/L/day astaxanthin in autotrophy and 2.6 to 3.1 mg/L/day in mixotrophy medium [14]. The biosynthesis of astaxanthin involves three molecules of isopentenyl pyrophosphate and one molecule of dimethylallyl pyrophosphate which are combined by IPP isomerase and converted to geranylgeranyl pyrophosphate by GGPP synthase. In algae, for example in *H. pluvialis *the biosynthesis of astaxanthin was observed in a stoichiometric fashion but the interaction mechanism was not clear. Studies on the molecular mechanism for the pathways showed that there was inter-dependence at the transcriptional level and the interaction was feedback-coordinated at the metabolite level. The biosynthesis took place in the endoplasmic reticulum, involving some diacylglycerol acyltransferases, which catalyzed astaxanthin esterification [15].

Glycogen and starch based metabolic pathways are responsible for the production of bioethanol in microalgae [16, 17]. Altering these pathways can help produce high quantities and types of bioethanol. For instance, a prokaryotic strain of *Synechococcus elongatus* PCC 7942 strain was engineered with *E. coli* alcohol dehydrogenase (encoded by y*qh*D gene) and ketoacid decarboxylase gene (*Kiv*D) for the production of isobutanol [18]. One another prokaryotic strain, *Synechococcus* sp. PCC6803 strain was also engineered by Varman et al. [19] by introducing two important genes, *Kiv*D and alcohol dehydrogenase (*adh*A gene), for the enhanced production of isobutanol.

Many industrially important chemicals and biopolymers are also produced by algae.For example, the cyanobacteria, *Synechococcus elongatus* UTEX 2973,was exploited for its fast growing nature and was expressed with *Cupriavidus necator* H16 derived heterologous *phaCAB* gene to enhance polyhydroxybutyrate (PHB) biopolymer production. The recombinant cyanobacteria had the capability to produce 420 mg L^–1^of PHB in 10 days cultivation period [20].

The studies discussed above show that many metabolic pathways in algae can be engineered or manipulated through the -omics approach to increase the production of industrially valuable products. There is a lack of both comprehensive and critical analysis of -omics applications with correlation to algal glycobiotechnology. Therefore, in this review aims to discuss various-omics approaches for strain improvements for higher productivity of desired commodities while highlighting the fate of algal glycobiology.

## Omics approaches

### Genomics

Advancement in omics technologies, metabolic engineering and system biology has pushed the idea of algal application as microbial cell factories to the forefront. Algae are highly diverse group of aquatic organisms and therefore produce diversity of bioproducts. Recent advances in genomics, proteomics and transcriptomics has made algae a “multi-use feedstock” with application in nutraceuticals, biofuel, material science and biomedical fields [21]. Genomics analysis provides information about biosynthetic and metabolic capabilities of algae and therefore gives a blueprint for enhancing its productivity as cell factories. Next generation sequencing technologies have made sequencing economic and reliable. Genome mining of algal strains has identified novel biosynthetic gene clusters for production of diverse compounds with application in biomedical and industrial microbiological arena [21]. Variation in gene and functionality can be examined by intra- and inter-species comparison using comparative genomics.

Algalomic technologies have been prominently used for biofuels. Algal oil particularly triacylglycerides are precursors for conversion into biodiesel and therefore algal lipids serving as feedstock for biodiesel are subjected to intensive investigation [22]. The first complete genome sequence was of model algae *Chlamydomonas reinhardtii* that was published in 2007 and subsequent sequencing of organellar and whole genome served as a base for functional annotation of lipid biosynthetic genes in microalgae [21]. Whole genome sequencing has helped in transferring knowledge of pathways and protein from animal, plant fungi and bacteria to algae. Adaptation of reference algae such as *P. tricornutum* and *C. reinhardtii* at genetic and molecular level has been experimentally characterized [22].

Development of a genetically engineered strain for higher efficiency in primary production and production of renewable energy requires comprehensive understanding of genetics and molecular biology. It has been pointed out that even though algae were utilized for the production of high value product; their greatest potential is in conversion of solar energy into chemical energy with low net carbon emission. Biocrude production from microalgae was established but was energetically expensive in comparison to conventional extraction of fossil fuels [23]. Although conventional strategies are available which could provide satisfactory result for strain improvement, genetic modification can provide rapid and substantial results for biocrude production. Incorporation of beneficial traits into producer strain requires comprehensive knowledge of algal biology which will help to conduct target optimization of the trait. It is important to understand that the algal gene regulation is also important to perform efficient and skillful manipulation. Library of algal genome has been expanded but is of limited use before systemic mapping, curation and annotation of genomes, as it consumes much more time than sequencing. Genome sequencing and analysis is important to understand and comprehend microalgal systems. Algae have huge diversity compared to which the available genome sequences are much smaller in number [23].

Many genetic tools have been added to the catalogue to improve the efficiency of algae for industrial purposes. Initial reports of genome editing in microalgae used Zinc Finger Nuclease for editing [24]. Diatom *Phaeodactylum tricornutum* was engineered using magnanuclease and transcription activator-like effector nucleases (TALEN) for enhancement in lipid accumulation. Gene UDP-glucose pyrophosphorylase was disrupted which resulted in 45 fold increase in accumulation of triacylglycerol, engineering of such enhanced lipid producing strain highlights the power of genome editing [25]. TALEN technique was used to knock out gene encoding for urease enzyme, PCR was used to detect knockout cassette in urease gene, western blot was used to detect absence of urease protein in mutant cell line and untargeted metabolomics was used to access the build-up of urea [26].

Genomics helps in predicting metabolism in algae and therefore reduces wet lab work. Information provided by genome sequencing can be employed in strategies for metabolic engineering. System biology giving leads for improvement in strain based on understanding of the metabolism, is dependent upon the data from omics-technology [27]. Flux balance analysis which is used to study the biochemical network requires reconstructed network based upon genome sequence data [27]. Breadth of functional capabilities of algae can be understood by ecological niche and phenotypic diversity of genome. Algae possess three different genomes, a large nuclear genome, a plastid genome and a mitochondrial genome [22]. Nevertheless, with the advent of new sequencing technologies, reduced cost and improved quality, the number of published algal genome sequences are escalating. Projects such as 10KP Genome Sequencing aims to sequence 3000 photosynthetic and non-photosynthetic protists and at least 1000 green algae. Genome sequencing can unravel evolution of algae and can provide mechanistic insight into adaptation [22].

### Transcriptomics

It is a molecular tool that provides insights into gene expression and to acquire the functional details of the microalgae strain. Transcriptomic analysis provides information about the actively encoding genomic region, molecular aspects, development and disease related genes [28]. Suppression subtractive hybridization (SSH) and serial analysis of gene expression (SAGE) are the techniques to study gene expression which does not require previous details of the genes under study. Prior information of the gene under study is essential during the sequencing tag (EST) study [29]. Transcriptomics studies were aimed to isolate total RNA from a sample of cell and determining abundance of each particular species of messenger RNA. These studies can be done by growing cells under different conditions which may aid in the addition of a gene to the gene set [30]. Databases for transcriptomic sequences do not provide data for untranscribed genomic region (such as promoter) and post transcriptional regulation but could serve in reconstruction of metabolic pathway and networks [31]. Analysis of transcriptome is done by generating expressed sequence tags which are formed by converting mRNA to cDNA and small fragments can be used to identify each DNA [30].

Functional annotation of green microalgae *B. braunii* race B, unraveled many operational biological pathways and the global comparisons revealed conservation of genome and transcriptome. Reconstruction of metabolic pathway and their participating enzyme for biosynthesis of terpenoid hydrocarbon in *B. braunii* strain Showa provided a metabolic and genetic framework for alterations, aimed towards increasing the production of hydrocarbons [31]. Induction of triacylglycerols (TAG) production in *Neochloris oleoabundans* and analyzing the expression of genes which are involved in TAG production at transcriptome level provided information about metabolic pathway which can be used further for detailed study and strain improvement for lipid accumulation. Quantification of expressed gene under nitrogen replete and nitrogen scarce condition and, assemblage of transcriptome in *N*. *oleoabundans* provided information about genes and pathway associated with production of lipid. This new repertoire of knowledge will enable metabolic engineering to aid in the production of sustainable liquid fuel [28]. *Chromochloriszo fingiensis*, industrially important algae, was involved in the synthesis of astaxanthin (a value added carotenoid) and lipid. *C. zofingiensis* synthesized and accumulated astaxanthin under different culture condition such as glucose induction, nitrogen deprivation, salt stress and high intensity light. A comparative study performed to evaluate the effect of different condition on astaxanthin synthesis showed that in comparison to other factors such as high intensity light and glucose induction, effect of nitrogen deprivation was more profound on astaxanthin synthesis. Transcriptome and time resolved caretenoid profiling delineated global response to cope with nitrogen deficient conditions. Reconstruction of carotenogenesis pathways revealed impairment of lutein biosynthesis, CO_2_ fixation, and stimulation of nitrogen metabolism and induction of astaxanthin synthesis leading to enhanced accumulation of astaxanthin [32]. Transcriptomic studies were performed to evaluate and improve the heat stress faced by *Dunaliella bardawil* (halophilic green algae). *D. bardawil* is a commercial strain used in the outdoor cultivation of β-carotene and the production is affected from heat during noon in hot summers. Transcriptomic analysis revealed that during heat stress, the genes for heat shock proteins and antioxidant enzymes were up-regulated. For energy production and survival, the strain shifted from aerobic to glycolytic metabolism. They suggested that altered lipid characteristics (chain length and unsaturation), ascorbate-glutathione cycle enrichment, and up-regulation of chloroplast membrane genes are vital for the thermotolerance of *D. bardawil* [33]. Transcriptomic evaluations can expedite the developments made toward using algae for biofuel generation. Unicellular green algae *Chlamydomonas moewusii* can produce hydrogen in both light and dark anaerobic conditions. RNA seq transcriptomic data for a time course was obtained giving insights about the expression pattern ofcontiguous involved in anaerobic fermentation, glycolysis, starch catabolism and hydrogen evolution in dark anaerobic condition. Analysis of expression of hydrogenase and fermentative pathway involved in balancing of redox reactions may be responsible for expression profile for hydrogenase activity and secretion of anaerobic metabolites. Both fundamental and applied research could take advantage of this cursory level of analysis [34]. These transcriptomic analyses provide background knowledge for the modification in algae at genomic level to increase specific production potential or tolerance to specific stresses.

### Proteomics

Genomics and transcriptomics provide details about the genomic complexity and the expression dynamics of a cell, whereas proteomics provide the information about the proteins maintain the structural, organizational and metabolic potential of the cell. Since the synthesis of active proteins involves multiple layers of regulation thus it is imperative to study the proteomic profile of a cell under a given condition [30]. The study of proteome (total protein content of cell) not only provides the description of activities within the cell but also about the biomarkers indicative of the status of a cell such as stress, apoptosis, production of any specific bioactive compound, and target for strain engineering etc [30, 35].

Oleaginous microalgae produce large quantities of TAG and fatty acids which serves as a feedstock for the production of bioproducts and biofuels. Guarnieri et al. utilized proteomic approach to study the mechanism and strain engineering targets in *Chlorella vulgaris* (oleaginous microalgae), for the overproduction of lipids required for increased biofuel production [35]. A careful examination of the proteome of this microalga in absence and presence of nitrogen revealed potential targets for strain engineering to obtain increased lipid content. The reason to this was attributed to the fact that in *C. vulgaris*, deprivation of nitrogen induces lipid accumulation. It was recorded that the fatty acid biosynthesis is induced in the absence of nitrogen along with a series of transcriptional factors, cell cycle and cell signaling regulators involved in lipid accumulation. Several of these were found in low-lipid state and thus were suggested as the targets for knockout or knockdown, whereas those found abundant during nitrogen deprivation can be considered for over expression [35]. The proteomic analysis on *Chlamydomonas reinhardtii* also suggested that the presence of regulatory framework lies in the fatty acid and TAG pathway during N deprivation [36, 37].

The first comparative proteomic study on wild type and mutant (for lipid over accumulation) *Tisochrysis lutea*, domesticated oleaginous algae, was performed by Garnier et al. [38]. The protein expression was studied during the absence and presence of nitrogen. The differential expression was recorded in correspondence to the two different metabolic conditions. They reported that 37 proteins were differentially expressed among the two strains, of which 17 proteins are linked with nitrogen starvation simultaneously with the accumulation of lipid. These proteins were identified to be involved in pathways such as carbohydrate, lipid, energy, pigment, and amino acid metabolisms, cell division, stress response, protein translation and photosynthesis. Two glycoside hydrolases, the coccolith scale protein and the plastid beta-ketoacyl-ACP reductase protein involved in carbohydrate catabolism, carbon homeostasis and fatty acid biosynthesis, respectively were found to have implications in lipid over-accumulation during starvation of nitrogen [38]. This study provides a detailed overview of the metabolism and new perspectives for lipid over-accumulation in *Tisochrysis lutea.* Proteomic analysis of *Chlamydomonas reinhardtii* was performed to get insights into the TAG biosynthesis and lipid droplet biogenesis [39]. *Chlamydomonas reinhardtii* is industrially important green algae and is unique in terms of its potential to simultaneously synthesize astaxanthin and TAG for storage in lipid droplet. It was reported that nitrogen deprivation induced seven lipid droplet proteins including l-gulonolactone oxidase, lipases, major lipid droplet protein, and caleosins. It was found that all these proteins when expressed in yeast had a high amount of TAG being formed. The expression of major lipid droplet protein was found to restore and enhance TAG production in wild-type *C. reinhardtii*, and for the first time it was identified that lipid droplet had an abundance of l-gulonolactone oxidase, which also facilitates the accumulation of TAG. This study provides a work plan to facilitate the improvement in TAG production through genetic engineering approach [39]. This suggests that quantitative data of translational proteomic analysis under diverse experimental conditions can fill the knowledge gap and provide understanding for pathways that differ at post-transcriptional level [24, 40]. Proteomics data along with the transcriptomics will provide the insights into microalgae and will serve as a foundation required for strain-improvement (Table 1).

**Table 1:** Microalgae strains along with the overview of reported omics studies for product formation

Organism	Omics approach	Conditions	Target product	References
*Auxenochlorella protothecoides*	Transcriptomics and proteomics	Effect of nitrogen, phosphorus and temperature starvation, and oil accumulation	Biofuel	[41, 42]
*Chlorella pyrenoidosa*	Transcriptomics and proteomics	Different nitrate levels and copper stress	Biofuel	[43, 44]
*Chlorella vulgaris*	Proteomics	Nitrogen deprivation	TAG and lipid	[35]
*Tisochrysis lutea*	Proteomics	Nitrogen starvation	Lipid accumulation	[38]
*Chromochloriszo fingiensis*	Transcriptomics and proteomics	Nitrogen deprivation and lipid droplet analysis	Fatty acids andcarotenoids	[32, 39]
*Dunaliella salina*	Transcriptomics and proteomics	Nitrogen depletion, oxidative stress, arsenate, salinity, and high bicarbonate ion level	Biofuel and glycerol	[24]
*Nannochloropsis gaditana*	Transcriptomics and proteomics	Nitrogen alterations and light intensity regimes	Biofuel	[45, 46]
*Picochlorum* sp.	Transcriptomics	High temperature and salinity stress	Biofuel	[24]

### Metabolomics

Microalgae contain numerous metabolites for which the properties and associations have not been fully identified because of the complexity of the metabolic processes of microalgae [47, 48]. Metabolomics focuses on analyzing metabolites and pathway in cells. Through metabolomics, a snapshot of the phenotype of microalgae can be obtained, which can be used to explain the mechanism of pathways. It is also possible to compare metabolites from wildtype and metabolites from mutant.

Metabolism studies require a high-speed and high-efficiency detection of all spectra that can be fully profiled to identify or quantify low-molecular metabolome. The most used analytical devices to date are Nuclear Magnetic Resonance (NMR) and Mass Spectroscopy (MS). In particular, the most used platforms in MS are LC-ESI (Electrospray ionization)-MS and MALDI (Matrix-assisted laser desorption/ionization)-TOF (Time-of-flight) MS, LC-ESI (Electrospray ionization)-MS. An ionization source is a place where samples are ionized in MS, divided into MALDI ionizing solid state samples and ESI ionizing liquid state samples. Both methods are widely used because they do not break down macromolecules and can be made into gas-state ions, which can be utilized for mass spectrometers. In metabolomics, Molecular Networking (MN) is used, which can analyze data collected through MS via identifying the spectrum of chemically related molecules based on similarities in patterns of molecular fragmentation [49].

Among the metabolomic techniques for microalgae, the following papers are summarized to study the products associated with glycobiotechnolgy (Table 2). Studies of *N*-glycosylation and *O*-glycosylation in microalgae have begun later than in other species. *N*-glycosylation pathway analysis of *Chlorella vulgaris* was first started in 2019 [50], and the *N*-glycosylation pathway analysis was initiated in 2013 for *Chlamydomonas reinhardtii*, a relatively well-known type of genetic information [51]. In the case of *Chlamydomonas reinhardtii*, a highly studied microalgae, there was further analysis of the *N*-glycosylation pathway [52] and it was investigated that the mechanisms of enzymes involved in xylosylation of *N*-glycan are like those of plants [53]. An analysis of the structure associated with *N*-glycan via MALDI-TOFMS was performed for *Porphyridium* sp. [54]. In addition, *Phaeodactylum tricornutum* is suitable to produce recombinant proteins through the analysis of mechanisms associated with *N*-glycan and biochemical characterization, and it was confirmed that the expression of specific genes increases glycoprotein production [55–57].

**Table 2:** Metabolomics studies of microalgae: application of glycobiotechnology

Strain	Analytical method	Target product	Key findings	References
*Chlorella vulgaris*	MALDI-TOF MS, LC-ESI-MS	*N*-glycans	Analysis of *N*-glycosylation pathway in *Chlorella vulgaris*	[50]
*Chlamydomonas reinhardti*	MALDI-TOF-MS	*N*-glycans	Analysis of *N*-glycosylation Pathway in *Chlamydomonas reinhardtii*	[51]
*Chlamydomonas reinhardtii*	LC-ESI-MS	*N*-glycans	Reexamination of the *N*-glycosylation pathway in *Chlamydomonas reinhardtii*	[52]
*Chlamydomonas reinhardtii*	LC-ESI-MS	*N*-glycans	An additional xylosyl transferase is connected with the xylosylation of protein *N*-linked glycans	[53]
*Phaeodactylum tricornutum*	MALDI-TOF–MS	*N*-glycans, LLO	Study of lipid-linked oligosaccharides	[55]
*Porphyridium *sp.	MALDI-TOF MS, MS/MS	*N*-glycans	The structural analysis of the *N*-glycans in the 66-kDa glycoprotein	[54]
*Phaeodactylum tricornutum*	LC-ESI-MS	*N*-glycans	Turning out recombinant proteins using *P. tricornutum *with high mannose-type *N*-glycans condition	[56]
*Phaeodactylum tricornutum*	MALDI-TOF/TOF	*N*-glycans	Over expression of the FuT54599 leads to an increase of the α (1,3)-fucosylation of the diatom endogenous glycoproteins	[57]
*Chlamydomonas reinhardtii*	ESI-MS	*O*-glycans	Analysis of the *O*-glycosylation pathway in *C. reinhardti*	[58]
*Chlamydomonas reinhardtii*	MALDI-TOF-MS. Ma	*O*-glycans	Genes encoding the serine *O*-α-galactosyltransferase were separated from *Chlamydomonas reinhardtii*	[59]
*Euglena gracilis*	LC-ESI-MS	*N*-glycans, *O*-glycans	*Euglena* has a complex glycan surface	[63]
*Chlorella vulgaris*	GCMS, LC-MS/MS and MN (Molecular networking)	Metabolite of *C.vulgaris* using metabolomics including glycolipid	Finding the presence of glycolipid via novel bioinformatics approaches such as the molecular networking	[60]
*Scytonema* sp. UIC 10,036, and *Nostoc* sp. UIC 10,110	LC-HRMS, XCMS Online	Primary metabolites (heterocyst glycolipids)	Lower nitrate levels inducing increased amounts of heterocyst glycolipids	[62]
*Chlorella* UMACC050	LC-MS analysis	Lipid (including glycolipid)	Lipid accumulation including glycolipid and enhancement of glycolytic activity induced by nitrogen depletion	[64]

The study of *O*-glycosylation through metabolomics is less active than that of *N*-glycosylation. *Chlamydomonas reinhardtii*, which has a lot of genetic information, has succeeded in researching the pathway of *O*-glycan and isolating it from Novel *O*-glycan [58, 59]. In addition, it was confirmed via metabolomics that the accumulation of lipids and glycol-lipid simultaneously increases due to nitrogen deficiency in microalgae [60–62]. Specifically, in the case of *Chlorella vulgaris*, the data obtained by MS was identified and analyzed through Molecular Networking (MN).

## Research challenges and future perspectives

Glycosylation is a type of posttranslational modification in which protein folding proceeds normally and is transferred to the correct site. The mechanism of glycosylation is well known in eukaryotes, such as mammals and plants. However, in microalgae, research on protein glycosylation has been insufficiently compared to glycolipid. Recently, some research has been done on the analysis of mechanisms for N-glycosylation. However, few mechanisms are known in the case of *O*-glycosylation [65]. Some studies are underway to investigate these relatively less well-known mechanisms and to increase the target product such as biomass, lipids, and value-added products at the same time.

Among many research approaches, the use of omics is in the spotlight because omics are used to elaborately identify their complex structuresand biological features in cells while considering the organism as a whole.Each of the omics is not mutually isolated; it is related and complementing. To get better understanding of characteristic of biomolecules, attempts have been made to integrate information from phenomics, genomics, transcriptomics, proteomics, and metabolomics [66]. Integrated analysis of omics helps to analyze extracts simultaneously to understand the whole system and biological changes caused by specific controls such as nitrogen-deficiency stress or mutations. Furthermore, it would also be possible to design a system to increase the production of the targeting material. Much of the integrated research is done in human and plant cells, but relatively little are done in microalgae [67, 68]. Nonetheless, this combination of omics would be an important and feasible tool for glycobiological products from microalgae.

In omics analysis, even when there is a lot of genetic analysis and proteomic data it could be also challenging to obtain meaningful information through genetic information. In other words, it is important to match genes and candidate proteins by combining information of gene, protein, and metabolite obtained from each omics. Because the amount of data obtained from high-throughput omics is voluminous, it is important to match DNA, RNA, protein, and metabolite obtained from the same sample accurately, without confusion [67]. Currently, analysis of glycobiotechnology using omics in microalgae is less studied than in other eukaryotes, and holds great potential to unlocking the glycobiology behind byproduct formation.

## **Conclusions**

Omics approaches seem highly promising for the manipulation of metabolic pathways of algal glycobiology to enhance the production of industrially important products. For instance, genomics can be used to understand the mechanistic function of algae that could facilitate enhanced glycolipids production. Transcriptomics can aid in reconstruction of innate metabolic pathways in algae to increase the production of hydrocarbons, pigments and triacyclglycerides. Expression of specific proteins associated with nitrogen deprivation with the help of proteomics has helped in the overproduction of glycolipids from algae. Metabolomics can help identify metabolites responsible for enhanced production of glycoproducts, non-invasively. Simultaneous analysis of the omic techniques and their interactions can provide a holistic understanding of the glycobiologial system of algae and products formed thereof. This aspect of algal glycobiotechnology is less explored and warrants investigation. Studies on the distribution of glyco conjugates such as lectin binding sites and lectin receptors at the algal cell surfaces would be highly relevant in this regard.

## Data Availability

Not applicable.
